# Pro-inflammatory cytokines and microRNAs in male infertility

**DOI:** 10.1007/s11033-021-06593-6

**Published:** 2021-07-28

**Authors:** Hana Attia, Federica Finocchi, Monia Orciani, Meriem Mehdi, Ines Zidi Jrah, Raffaella Lazzarini, Giancarlo Balercia, Monica Mattioli Belmonte

**Affiliations:** 1 Department of Histology Embryology and Cytogenetic, Faculty of Medicine, University of Monastir, Monastir, Tunisia; 2 Laboratory of Cytogenetics and Reproductive Biology, Center of Maternity and Neonatology, Fattouma Bourguiba University Teaching Hospital, Monastir, Tunisia; 3 Division of Endocrinology, Department of Clinical and Molecular Science (DISCLIMO), Polytechnic University of Marche, Ancona, Italy; 4 Laboratory of Histology, Department of Clinical and Molecular Science, Polytechnic University of Marche, Ancona, Italy

**Keywords:** Male infertility, TNF-α, IL-6, IL-1α, MicroRNAs

## Abstract

**Background:**

Male infertility is a problem that affects 10–15% of men of reproductive age. In particular, gametogenesis is a complex process in which inflammation may play a central role through the secretion of cytokines and the expression of microRNAs. We assessed the potential role of proinflammatory cytokines (TNF-α, IL-6 and IL-1α) and microRNAs (miR-146a-5p, miR-34a-5p and miR-23a-3p) in the seminal plasma of infertile men compared to controls, evaluating their correlation with seminal and biochemical parameters.

**Methods and results:**

Expression of cytokines and microRNAs was analyzed by ELISA and q-PCR. Our data shows that IL-1α was significantly increased in the azoospermic group compared to controls, TNF-α mRNA was more expressed in the oligozoospermic group than controls. There were no significant differences in miRNAs expression among the three groups. The correlations between sperm parameters and inflammatory markers were evaluated, however no significance was highlighted.

**Conclusions:**

The determination of each inflammatory marker separately in the seminal plasma of subfertile men, despite some significant differences, does not have a diagnostic value in male infertility even if an assay of selective pro-inflammatory cytokines and microRNAs in the semen may improve the diagnosis of male infertility.

## Introduction

Spermatogenesis is a very complex, highly organized process and its disruption may lead to male infertility. The achievement of male fertility requires the integration, communication, and functionality of distinct cell types in the testis throughout germ cell maturation [1]. Moreover, it is tightly controlled by cytokines [2] and their level has been found dysregulated in the seminal plasma of men with hypo-fertility [2]. Among the others, TNF-α is a crucial molecule that stimulates sperm peroxidation by the rise of reactive oxygen species (ROS) generation and apoptosis processes [3]. Cytokines are released by different immune cells in the male genital tract, such as macrophages, monocytes, lymphocytes, dendritic cells, and also as a reaction to foreign antigens and pathogens within chronic inflammation [4]. Several cytokines, including IL-1α, IL-6, TNF-α, and activin A, are produced in a cyclical way by the Sertoli or spermatogenic cells during the maturation cycles of the seminiferous epithelium, implying that their actions are crucial to controlling this basic aspect of testicular functions [5]. Furthermore, it has been establish that exposing spermatozoa to elevated concentrations of TNF-α arise in a significant loss of their genomic integrity and functionality [6, 7]. The relationship between pro-inflammatory cytokines, including IL-1α and IL-6, and semen quality, is still debated. Some authors found a link between ILs and sperm motility/ova-penetrating ability of spermatozoa [8], others reported no significant differences in ILs [9], potentially as a result of different population characteristics and assay methods.

Besides cytokines, other molecules have an important role in male infertility [10]. Khawar et al. reported as the aberrant expression of miRNAs influent spermatogenesis at numerous phases and in different cell types, frequently resulting in infertility [11]. Above all, the function of some miRNAs seems to be essential for male fertility. The expression of miR-146a-5p is finely regulated during spermatogenesis: it is strongly expressed in undifferentiated spermatogonia whereas its transcription is strongly decreased in differentiating spermatogonia [12]. The miR-23 family (miR‐23a, b, and c) is strongly reduced in nemaspermic cell and seminal plasma exosomes of subfertile vs. fertile men. The miR‐23 family regulates some spermatogenesis‐specific genes such as PFKFB4, HMMR and SPATA6 [13]. Studies have shown that semen parameters (sperm count, motility and morphology) negatively correlated with the expression of the miR-23 family, while they positively correlated with reduced expression of the mentioned genes. Hence, a role of the miR-23 family in the pathogenesis of male infertility is hypothesized [13, 14].

In the current study, we aimed to evaluate the gene expression levels of pro-inflammatory cytokines (IL-6, TNF-α) and miRNAs (miR-146a-5p, miR-34a-5p and miR-23a-5p) and the protein expression of IL-1α both in infertile patients and controls, to clarify their interdependent relationship and their role in the etiopathology of male infertility.

## Materials and methods

### Study population and sample collection

We selected 45 patients who were referred to the Fattouma Bourguiba University Teaching Hospital of Monastir’s fertility clinic. The study included 15 azoospermic patients, 15 oligozoospermic patients and 15 healthy normozoospermic men as controls. Blood and sperm samples were taken from each patient. The study was approved by Fattouma Bourguiba University Teaching Hospital of Monastir ethics committee, all patients had given their consent to participate in the study.

All patients included in the study had undergone the analysis during routine andrological screening. Exclusion criteria were endocrine disorders, urogenital tract infections, cryptorchidism, testicular cancer, or previous chemo/radiotherapy.

All patients had a typical karyotype, and five of the oligozoospermic patients had a varicocele. Furthermore, there was no presence of Y chromosome microdeletions, obstructions, or genetic causes for all azoospermic patients (e.g. Klinefelter).

The semen examination was carried out according to WHO 2010 guidelines [15] and viability was also assessed by Eosin Y. In cases of azoospermia, the diagnosis was confirmed on a second semen sample.

To evaluate sperm morphology the seminal samples were stained by the kit spermoscan (Fast staining kit for spermocytograms, Martillac-France).

To provide a distribution of morphological abnormalities, morphological assessment according to the updated David classification was also performed [16].

Seminal plasma was obtained by centrifuging the sample at 13,000 rpm for 15 min and frozen at − 20 °C for subsequent analyzes.

### Hormonal and metabolic analysis

A peripheral blood sample was taken from each patient in the morning after overnight fasting. Serum levels of follicle-stimulating hormone (FSH), luteinizing hormone (LH), prolactine and estrogen hormones were measured by Radioimmunoassay. In our laboratory, normal ranges for adults were 10 mU/mL (FSH), 1.1–10 mU/mL (LH), and 50–250 pmol/L (prolactine) and 20 ng/mL (estrogen). We also evaluated the lipic profile of the patients through triglycerides, (TG; normal value < 1.8 mmol/L) and total cholesterol (CH; normal value < 5.2 mmol/L).

### ELISA

IL-1α concentration was determined with ELISArray kit (Biorbyt, Cambridge, United Kingdom) and the results were expressed in picograms per milliliter in accordance with the manufacturer’s instructions.

### RNA extraction

Total RNA was extracted from 300 µl of the seminal plasma with Complete RNA Purification Kit (Norgen Biotek Company, Thorold, ON, Canada) according to the manufacturer’s instructions. The RNA was kept at − 80 °C until it was used.

### Quantitative RT-PCR miRNAs

MiRNAs were quantified using TaqMan MicroRNA Assay hsa-miR-146a-5p, hsa-miR-34a-5p and hsa-miR-23a-5p, with the endogenous control U6 snRNA for the seminal plasma (Thermo Fisher, Milan, Italy) as previously described [17–19].

Fluorescence data were converted to cycle threshold (Ct) by ExpressionSuite v1.0.4 (Applied Biosystems) and the quantity for each target miRNA was calculated by the ΔCt method normalized on the basis of the U6 snRNA. Undetermined values were considered as equal to the maximum number of cycles, namely 35.

### Expression of IL-6 and TNF-α by real-time PCR

Real-time PCR was carried out using an Eppendorf Master Cycle (Hamburg, Germany) instrument and EVA Green PCR Master Mix (Bio-Rad, Milan, Italy) according to the manufacturer’s instructions. At the end of the amplification process, a melting stage was added. There was no non-specific amplification as determined by the melting curve. All samples were tested in triplicate and β-actin was used as reference genes for data normalization for TNF-α and IL-6. The quantity for each target mRNA was calculated by the ΔCt method normalized on the basis of the β-actin and RPL30. The primer sequences were as follows:

- TNF-α for: 5′-GGTGCTTGTTCCTCAGCCTC-3′
  rev: 5′-AGATGATCTGACCTGCCTGGG-3′
- IL-6 for: 5′-ATTCTGCGCAGCTTTAAGGA-3′
  rev: 5′-AACAACAATCTGAGGTCGCC-3′
- β-actin for: 5′-GGACTTCGAGCAAGAGATGG-3′
  rev: 5′-GATGGAGTTGAAGGTAGTTTCG-3′

### Statistical analysis

The statistical analysis was carried out with the Statistical Package for the Social Sciences (SPSS) 23.0 software (SPSS Inc., Chicago, IL, USA). In case of normal distribution, data were presented as mean ± SD; one-way analysis of variance (ANOVA), Kruskal Wallis, and Mann–Whitney tests were carried out to compare control and patient groups (both azoospermic and oligozoospermic patients). The coefficients of Spearman’s correlation were also determined. Values of p < 0.05 were considered statistically significant.

## Results

### Semen analysis

The study population included 45 subjects, of whom 15 had azoospermia, 15 had oligozoospermia and 15 were normozoospermic. All subjects underwent semen analysis.

There were no statistically significant differences among the age of azoospermia and oligozoospermia vs. controls (37 ± 5.4; 33 ± 4.8, and 34 ± 5.2 years, respectively (p > 0.24)). Seminal parameters assessment for the three groups of subjects has been reported in Table ﻿1.

**Table 1:** Mean ± SD for age and semen parameters of the three groups

	Control (n = 15)	Oligozoospermia (n = 15)	Azoospermia (n = 15)	p-value^1^	p-value^2^	p-value^3^
Age (years)	34 ± 5.2	33 ± 4.8	37 ± 5.4	NS	NS	NS
Volume (ml)	3.3 ± 1.30	3.86 ± 1.21	3.39 ± 0.20	NS	NS	NS
pH	7.8 ± 0.16	7.94 ± 0.12	7.95 ± 0.02	NS	NS	NS
Concentration (× 10^6^spz/ml)	181.45 ± 116.8	8.43 ± 6.4	–	**0﻿**﻿.0﻿1﻿*	–	–
Leucocyte (× 10^6^/ml)	0.98 ± 1.65	0.90 ± 0.59	0.67 ± 0.04	NS	NS	NS
Progressive motility (%)	48 ± 9.55	26.15 ± 19.27	–	**0**.02*	–	–
Vitality (%)	18.60 ± 13.62	20.33 ± 10.26	–	NS	–	–
Abnormal forms (AF, %)	81.33 ± 4.38	90.53 ± 6.65	–	**0**.01*	–	–
Multiple anomalies index (IAM)	1.58 ± 0.15	1.85 ± 0.27	–	**0**.02*	–	–

Bold values indicate the statistically significant difference

Parametric data was analyzed by Anova test. NS: p-value Not significant; p-value^1^: control vs. oligozoopermia; p-value^2^: control vs. azoospermia; p-value^3^: comparison between three groups

*Significant difference p < 0.05

Oligo- and azoospermic patients showed some differences compared to control subjects: the total motility was reduced in oligozoospermic patients (26.15 ± 19.27%) compared to controls (48 ± 9.55%) (p = 0.02), whereas the percentage of the spermatozoa with abnormal forms (AF) and the multiple anomalies indexes (IAM) were higher in oligozoospermic patients than in controls (90.53 ± 6.65 and 81.33 ± 4.38% for AF, p = 0.01; 1.85 ± 0.27; 1.58 ± 0.15 % for IAM, p = 0.02).

### Hormonal and metabolic parameters

The dosage of LH, prolactin, TG, and CH showed no significant variation among the three groups. Only FSH and eostrogen were significantly higher (p = 0.02) in azoospermic patients than in controls and oligozoospermics (Table 2).

**Table 2:** Mean ± SD of serum hormones between control and study groups

	Control (n = 15)	Oligozoospermia (n = 15)	Azoospermia (n = 15)	p-value^1^	p-value^2^	p-value^3^
LH (mU/ml)	4.51 ± 1.64	5.69 ± 3.52	6.31 ± 5.87	NS	NS	NS
FSH (mU/ml)	3.51 ± 1.09	9.82 ± 9.06	16.80 ± 12.90	NS	0.01	**0**.02*
Estrogen (ng/ml)	21.38 ± 1.09	24.85 ± 7.59	44.27 ± 21.25	NS	0.01	**0**.02*
Prolactin (pmol/l)	142.62 ± 86	163.05 ± 76	221.20 ± 103	NS	NS	NS
Triglycerides (mmol/l)	4.43 ± 0.88	3.62 ± 1.03	4.20 ± 0.71	NS	NS	NS
Cholesterol (mmol/l)	1.09 ± 0.71	1.23 ± 0.45	1.74 ± 1.07	NS	NS	NS

Bold values indicate the statistically significant difference

Parametric data was analyzed by Anova test. NS: p-value Not significant; p-value^1^: control vs. oligozoospermia; p-value^2^: control vs. azoospermia; p-value^3^: comparison between 3 groups

*Significant difference with control group (p < 0.05)

### Expression of inflammatory biomarkers and correlation with semen characteristics and biochemical parameters

In seminal plasma mRNA of cytokines (TNF-α, IL-6), miRNAs (miR-146a-5p; miR-34a-5p; miR-23a-5p) and protein expression of and IL-1α have been detected, all the data are shown in Table 3.

**Table 3:** Mean ± SD and median (in brackets) of pro-inflammatory cytokines and microRNA in control, oligo- and azoospermic groups analyzed using Anova, Kruskal Wallis and Mann–Whitney test

	Control	Oligozoospermia	Azoospermia	p-value^1^	p-value^2^	p-value^3^
TNF-α	0.056 ± 0.079 (0.021) (n = 10)	0.336 ± 0.567 (0.105) (n = 15)	0.168 ± 0.243 (0.067) (n = 11)	**0**.05*	NS	NS
IL-6	0.001 ± 0.0004 (0.0010) (n = 11)	0.018 ± 0.056 (0.0014) (n = 14)	0.005 ± 0.012 (0.0015) (n = 10)	NS	NS	NS
MiR-146a-5p	0.048 ± 0.06 (0.0218) (n = 10)	0.059 ± 0.011 (0.228) (n = 10)	0.048 ± 0.046 (0.041) (n = 10)	NS	NS	NS
MiR-34a-5p	0.033 ± 0.055 (0.009) (n = 10)	0.015 ± 0.010 (0.014) (n = 10)	0.061 ± 0.071 (0.032) (n = 10)	NS	NS	NS
MiR-23a-3p	0.006 ± 0.011 (0.0012) (n = 10)	0.0026 ± 0.0020 (0.0021) (n = 10)	0.011 ± 0.010 (0.012) (n = 10)	NS	NS	NS
IL-1α (pg/ml)	12.43 ± 8.03 (12.92) (n = 14)	11.01 ± 10.64 (4.52) (n = 13)	23.33 ± 18.35 (21.16) (n = 13)	NS	**0**.05*	**0**.05*

Bold values indicate the statistically significant difference

TNF-α, IL-6, miR-0146a-5p, miR-34a-5p and miR-23a-3p are analyzed by Real-Time PCR; IL-1α analyzed by ELISA

p-value^1^: control vs. oligozoospermia; p-value^2^: control vs. azoospermia; p-value^3^: comparison between three groups

*Significant difference with control group (p ≤ 0.05)

Among the three cytokines, IL-1α showed a statistically significant variation in azoospermic group compared to controls (p < 0.05) while TNF-α mRNA was more expressed in the oligozoospermic group compared to controls (p < 0.05). For miRNAs, no significant differences among the three groups were detected (Table 3).

The associations between miRNAs and cytokines expression in the three groups (control, oligo- and azoospermic) were checked. For oligozoospermic, only a positive correlation between miR-23a-3p and miR-34a-5p (r = 0.576; p = 0.008) compared to controls was found.

Correlations among semen parameters, miRNAs and inflammatory markers were evaluated in oligo- and azoospermic groups vs. controls. For the oligozoospermic group, positive correlations were found between TNF-α vs. viability (r = 0.456, p = 0.022) and FSH (r = 0.476, p = 0.025); IL-6 shows a positive correlation vs. the percentage of abnormal form (r = 0.571, p = 0.003). Levels of CH correlate negatively with IL-1α (r = − 0.485, p = 0.026), miR-146a-5p (r = − 0.478, p = 0.045) and miR-23a-3p (r = − 0.524, p = 0.026). Comparing azoospermic groups to controls, a negative correlation between IL-6 vs. TNF-α (r = − 0,496; p = 0.036) was found; in addition, a positive correlation was noted between IL-1α and miR-146a-5p (r = 0.513; p = 0.025) as well as between miR-146a-5p and miR-23a-3p (r = 0.466; p = 0.044). Lastly, a negative correlation between IL-1α vs. semen volume (r = − 0.515, p = 0.006) and IL-6 vs. CH (r = − 0.567, p = 0.028) was found.

## Discussion

In the current research, IL-1α protein expression, IL-6 and TNF-α gene expression, and miRNAs expression (miR-146a-5p, miR-23a-3p, miR-34a-5p) were evaluated, along with their association with sperm parameters in control, oligo- and azoospermic patients.

Concerning the inflammatory markers, a previous study highlighted that the oligozoospermic group had higher significant concentrations of IL-6 and the azoospermic group had higher significant concentrations of TNF-α [20]. However, the authors analyzed the protein and not the mRNA expression. The other differences in semen parameters such as motility, abnormal form, and IAM detected between control and oligozoospermic groups, confirm what was previously reported by Agarwal et al. [21].

Higher levels of FSH and estrogen have been detected in azoospermic patients compared to controls. These results are in line with previous researches reporting as an elevated concentration of these hormones correlates with impaired spermatogenesis and fertility [22].

After semen characterization, the expression of cytokines (IL-6, IL-1α, TNF-α) and miRNAs quantification (miR-34a-5p, miR-146a-5p, miR-23a-3p) was evaluated.

Among cytokines, TNF-α was upregulated in oligozoospermic patients vs. controls and azoospermics. TNF-α is the most studied and effective molecule in germ cell apoptosis, peritubular cell secretion, and spermatogenesis control [23]. In Sertoli and Leydig cells are present TNF-α receptors, allowing it to control secretion from these cells [24, 25]. Some studies have shown a negative association of TNF-α plasma levels with sperm motility and morphology [26, 27]. This upregulation seems to contradict results from Chyra-Jach et al. reporting a down-regulation of about 10% in the secretion of TNF-α in oligozoospermic vs. control [28]. Nevertheless, they measured the protein amount and not the mRNA expression therefore this apparent discrepancy may imply a post-transcriptional control.

Besides, the expression of TNF-α in the oligozoospermic group correlates with a higher level of FSH but it is not associated with all the other clinically relevant parameters of semen quality, confirming results by Eggert-Kruse et al. [29], although the TNF-α receptor in Sertoli cells is under control by the FSH hormone [24].

IL-1α secretion was significantly higher in azoospermic patients than in controls and oligozoospermic. IL-1α is primarily developed by the seminiferous epithelium, where it has been identified as a potent growth factor for immature Sertoli cells as well as spermatogonia in numerous studies [30, 31].

As demonstrated by Buch et al. cytokines such as IL-1α and TNF-α can lead to sperm damage through the production of ROS and subsequent lipid peroxidation [32, 33].

IL-6 expression was almost the same in the three groups in line with results from Chyra-Jach et al. [28]. The association between IL-6 cytokine levels and semen quality is still debated, on one hand, elevated levels of IL-6 have been documented in infertile men with oligo-astheno-teratozoospermia, on the other hand, there is no linkage between cytokine levels and sperm quality [33–35]. The expression of miR-146a-5p, miR-34a-5p, miR-23a-3p did not exhibit significant changes between infertile men and controls; nevertheless, in oligozoospermic and in an azoospermic group compared to controls, miR34a-5p positively correlated with miR-23a-5p and since each of them is involved in male infertility, this positive correlation may reflect a synergistic action. It is important to remember that the members of miR-34 family are regulated directly by p53 and their upregulation stimulates the arrest of the cell cycle in stage G1 and induces apoptosis [36].

In the azoospermic group compared to controls, miR-146a-5p positively correlates with IL-1α but not with the TNF-α pathway as reported by others previously [37, 38]. MiR-146a-5p has been shown to have anti-inflammatory properties, in fact it regulates IL-1 receptor-associated kinase (IRAK)-1 and TNF receptor-associated factor (TRAF)-6; these are mediators of the cellular response to IL-1α [39, 40].

As a final point, correlations between inflammatory markers and semen parameters were checked; for oligozoospermic patients compared to controls, IL-6 positively correlates with the percentage of an atypical form of spermatozoa, according to previous results reporting as high IL-6 concentration occurred significantly in case of reduced functional competence of spermatozoa [34]. Interestingly, cholesterol negatively correlates with three inflammatory markers (IL-6, IL-1α, miR-23a-3p); testicular functions such as steroidogenesis, Sertoli cell function, and germ cell differentiation all include cholesterol homeostasis [41, 42]. The decrease of cholesterol together with an increase of inflammatory markers may synergically impact the reproductive function leading to male infertility. The same negative correlation between cholesterol and IL-6 was also observed in azoospermic patients; in this group, no particular other correlation was found confirming as cytokine concentrations are not always related to sperm parameters [42–45].

Thus, even though an assay of selective cytokines and microRNAs in the semen can improve the diagnosis of male infertility, measuring each inflammatory marker separately in the seminal plasma of subfertile men has no diagnostic value in male infertility, despite some important differences. In light of the small number of studies that have investigated the relationship between pro-inflammatory cytokines, miRNAs and sperm parameters, ours may represent an intriguing approach. However, to finer comprehend the mechanisms that provide pathways for miRNAs up or downregulation, as well as the function of pro-inflammatory cytokines in seminal plasma and male infertility, further studies with larger numbers of patients are needed.

## Conclusions

In summary, in fertile and infertile patients’s seminal plasma, the expression of cytokines (TNF-α, IL-6), miRNAs (miR-146a-5p, miR-34a-5p, miR-23a-3p) and IL-1α protein was not strongly correlated to any standard sperm parameters. Since the seminal fluid is consisting of the different secretions coming from the testis, epididymis, prostate and accessory glands, it is difficult to identify the direct origins of the cytokines and miRNAs analyzed. Cytokines work within a network that can be influenced by various factors, therefore the levels of expression observed in the study may have been influenced by poor selection and processing of the samples as well as by their molecular instability. Hence, proinflammatory cytokines appear neither to play an essential role in male infertility nor to contribute to oligospermia and azoospermia. Cytokines may activate inflammation directly or indirectly in the presence of other molecules such as miRNAs, hormones, lipidic molecules. More research in seminal plasma is needed to improve our knowledge about the key role of pro-inflammatory cytokines and microRNAs. It is also important to underline that the analysis of the expression of the IL-1α protein provides different information compared to the analysis of the mRNA expression of the other two cytokines, therefore it will be important to also evaluate the protein expression of the other molecules.

The investigation of the relationships between pro-inflammatory cytokines, specific miRNAs, and biochemical molecules in seminal plasma may give new insights about inflammation and/or dysfunction of spermatogenesis in hypo-fertile patients.
